# Complete Genome Sequence of Cluster O Mycobacterium smegmatis Bacteriophage Ryadel

**DOI:** 10.1128/MRA.01594-18

**Published:** 2019-03-28

**Authors:** Travis Miller, Danielle Bachhofer, Ashleigh Cooper, Jessica Doty, Josh Katuri, Jonathan Musgrave, Aleksey Palumbo, Heidi Spann, Amanda Stone, Keith Emmert, Julie Edwards, Jesse Meik, James Pierce, Dustin Edwards

**Affiliations:** a Department of Biological Sciences, Tarleton State University, Stephenville, Texas, USA; b Department of Mathematics, Tarleton State University, Stephenville, Texas, USA; KU Leuven

## Abstract

Mycobacteriophage Ryadel is a newly isolated cluster O *Siphoviridae* bacteriophage, characterized by an unusual prolate capsid, containing a 72,658-base-pair double-stranded DNA genome with 132 predicted protein-coding genes. Conserved among cluster O bacteriophages, the Ryadel genome contains 31 copies of a unique 17-bp sequence with dyad symmetry.

## ANNOUNCEMENT

**FIG 1:**
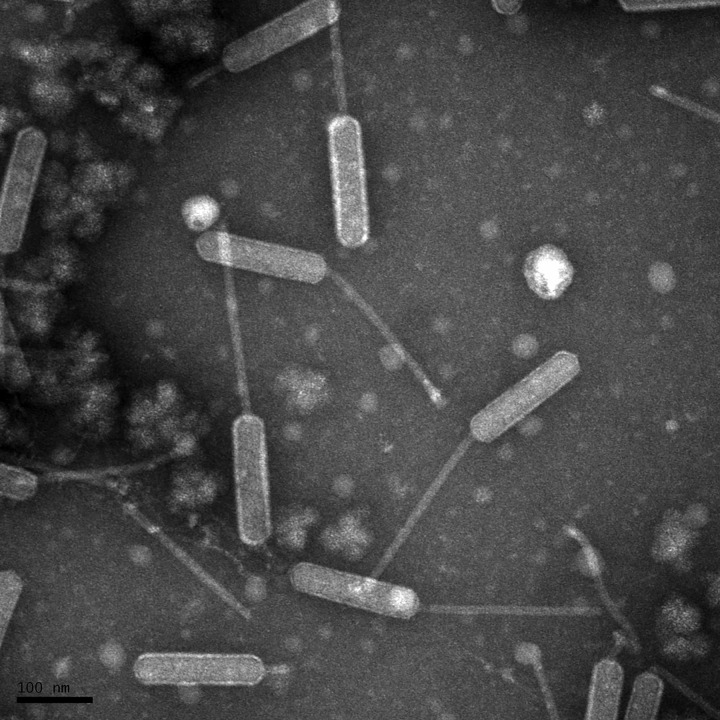
Transmission electron microscopy (TEM) of mycobacteriophage Ryadel. Purified high-titer lysate was placed on a carbon type-B 300 mesh grid, stained with uranyl acetate, and imaged by a FEI Tecnai G^2^ Spirit BioTWIN (NL1.160G). TEM micrographs of negatively stained mycobacteriophage Ryadel showed a prolate capsid that was 160 nm in length by 40 nm in width (4:1 ratio), a morphology that corresponds to that of members of cluster O bacteriophages within the *Siphoviridae* family.

Mycobacteriophage Ryadel was isolated from sandy soil samples collected beneath a rotting hay bale in Stephenville, Texas (32°16′43.601″N, −98°08′52.101″W). Soil samples were placed in 7H9 liquid medium, and the supernatant was passed through a 0.22-µm filter for direct isolation of bacteriophages. Filtered supernatant was incubated with Mycobacterium smegmatis mc^2^155 at 37°C for 48 hours and resulted in small-sized lytic plaques. Ryadel was purified by collecting virus from well-isolated plaques from the direct isolation and two successive rounds of serial dilutions. Negative-staining transmission electron microscopy (Fig. 1) of isolated mycobacteriophage Ryadel displayed siphoviral morphology with an unusual prolate capsid that was 160 nm in length by 40 nm in width (4:1 ratio), which is characteristic of cluster O bacteriophages (1).

A Promega Wizard DNA clean-up kit was used to isolate DNA from the purified bacteriophage. The Pittsburgh Bacteriophage Institute prepared a Genomic DNA sequencing library using the NEB Ultra II kit, which was run on an Illumina MiSeq instrument with 47 other samples, yielding ∼172,400 single-end 150-base-pair Ryadel reads representing ∼325-fold genome coverage. A single bacteriophage contig was assembled from raw reads using Newbler 2.9 with default settings, and it was checked for completeness, accuracy, and genome termini using Consed 29.0 (2, 3). The virus contains a double-stranded DNA genome that is 72,658 base pairs in length with a G+C content of 65.2%. The accumulation of aligned reads indicated a linear genome with a 3′ single-stranded terminal overhang of 5′-GTGT-3′. NCBI BLASTn (https://blast.ncbi.nlm.nih.gov/) whole-genome alignment (4) showed 98% nucleotide identity to the cluster O mycobacteriophages Familton (GenBank accession number MG099943) and Catdawg (GenBank accession number KF017002) (5). Characteristic of cluster O bacteriophages, the Ryadel genome contains 31 copies of a unique 17-bp sequence with dyad symmetry consisting of a 7-bp inverted repeat separated by 3 bp (5′-TGTTCGGNNNCCGAACA-3′) (1). This repeat does not occur in the genomes of Mycobacterium tuberculosis or Mycobacterium smegmatis mc^2^155; however, there are two copies present in the genome of *Mycobacterium* sp. 05-1390 (1).

Glimmer v3.02 (6, 7) and Genemark v2.5p (8, 9) were used to autoannotate the genome. Manual inspection included refinement of start sites and annotation revision using Phamerator (https://phamerator.org/) (10), DNA Master v5.23.2 (http://phagesdb.org/DNAMaster/), and PECAAN (https://pecaan.kbrinsgd.org/). Mycobacteriophage Ryadel was predicted to contain 132 protein-coding genes, and no tRNA genes were identified by ARAGORN v1.2.38 (11) or tRNAscan-SE v2.0 (12). Putative functions of 34 (25.8%) of 132 predicted protein-coding genes were assigned using HHpred v3.0beta (13, 14) and NCBI BLASTp (4). Similar to other cluster O bacteriophages, the Ryadel genome is mostly arranged in three transcriptional blocks and contains nine strongly predicted SigA-like promoters (5′-TGTCAA–17 bp–TGAAT-3′) (1). Leftward-transcribed genes 1 to 13 (6.5% of genome) encode endonuclease VI and DNA methylases. Rightward-transcribed genes 14 to 76 (58.5% of genome) encode virion structural and assembly proteins, DNA primase/polymerase, HNH endonucleases, *O*-methyltransferase, glycosyltransferases, d-Ala-d-Ala carboxypeptidase, a lysis cassette containing lysin A and lysin B, and holin proteins. Leftward-transcribed genes 77 to 132 (35% of genome) encode DNA polymerase III sliding clamp beta, Ku-like double-stranded DNA (dsDNA) break-binding protein, and ParB-like dsDNA partitioning protein.

### Data availability.

The mycobacteriophage Ryadel genome is available at GenBank under accession number MH590592. Raw reads are available in the SRA under accession number SRX4721442.
